# Artificial Intelligence Education for the Health Workforce: Expert Survey of Approaches and Needs

**DOI:** 10.2196/35223

**Published:** 2022-04-04

**Authors:** Kathleen Gray, John Slavotinek, Gerardo Luis Dimaguila, Dawn Choo

**Affiliations:** 1 Centre for Digital Transformation of Health The University of Melbourne Parkville Australia; 2 South Australia Medical Imaging Flinders Medical Centre Bedford Park Australia; 3 College of Medicine and Public Health Flinders University Adelaide Australia; 4 Murdoch Children's Research Institute Royal Children's Hospital Parkville Australia

**Keywords:** artificial intelligence, curriculum, ethics, human-computer interaction, interprofessional education, machine learning, natural language processing, professional development, robotics

## Abstract

**Background:**

The preparation of the current and future health workforce for the possibility of using artificial intelligence (AI) in health care is a growing concern as AI applications emerge in various care settings and specializations. At present, there is no obvious consensus among educators about what needs to be learned or how this learning may be supported or assessed.

**Objective:**

Our study aims to explore health care education experts’ ideas and plans for preparing the health workforce to work with AI and identify critical gaps in curriculum and educational resources across a national health care system.

**Methods:**

A survey canvassed expert views on AI education for the health workforce in terms of educational strategies, subject matter priorities, meaningful learning activities, desired attitudes, and skills. A total of 39 senior people from different health workforce subgroups across Australia provided ratings and free-text responses in late 2020.

**Results:**

The responses highlighted the importance of education on ethical implications, suitability of large data sets for use in AI clinical applications, principles of machine learning, and specific diagnosis and treatment applications of AI as well as alterations to cognitive load during clinical work and the interaction between humans and machines in clinical settings. Respondents also outlined barriers to implementation, such as lack of governance structures and processes, resource constraints, and cultural adjustment.

**Conclusions:**

Further work around the world of the kind reported in this survey can assist educators and education authorities who are responsible for preparing the health workforce to minimize the risks and realize the benefits of implementing AI in health care.

## Introduction

### Background

Artificial intelligence (AI) is widely expected to have broad and deep impacts on health care. In the past few years, several books have appeared on this topic whose covers connect it variously with business success, creative destruction, robotic assistance, care that is more human, and treatment that is more precise. Dozens of review papers have synthesized the growing body of scientific literature focusing on applications in an array of sociotechnical factors and care specializations: aged care, decision support, efficiencies, emergency medicine, ethics, nursing, pathology, psychiatry, and workflows.

Across the board, a key consideration is how to prepare the current and future health workforce for the possibility of using AI in health care. The World Health Organization and many national health systems around the world have flagged the importance of a health workforce that understands how to work properly with AI [1]. However, there is no straightforward answer to the question of how to provide education and professional development that prepares the health workforce adequately to do so (this topic is not to be confused with the use of AI-supported teaching techniques and learning analytics tools in health professions education, as reviewed by Hasan Sapci and Aylin Sapci [2]).

Disproportionately few peer-reviewed papers have been written on this aspect of AI in health care. Those extant studies focus chiefly on medical professionals [3-10]; there is scant reference to other health professions (eg, nursing [11]) or the health workforce broadly [12,13]. Almost all of them are commentaries by individuals advocating for appropriate education and development. A few papers are based on surveying the attitudes or knowledge of current medical students about AI [14], and one paper even offers a way to measure medical students’ readiness to learn about AI [15]. At present, there is no obvious consensus among educators about what needs to be learned or how this learning may be supported or assessed.

In Australia, although medical specialist colleges such as radiology, dermatology, and ophthalmology are developing AI competencies and training packages for their members, most people in the health sector whose work will be affected by the increased use of AI have minimal access to relevant education or professional development. The Australian Alliance for Artificial Intelligence in Healthcare—a nonprofit network of >100 partners and stakeholders in academia, government, consumer, clinical, industry organizations, and peak bodies seeking to translate AI technologies into health services [16]—offered a way to consult widely about how to approach AI education and development for a national health workforce. The Alliance’s Workforce Working Group, convened by KG and JS, gave rise to a project to gather and share information on how educational authorities are preparing Australia’s health workforce to work with AI and what gaps in curriculum and educational resources may need attention.

### Objective

The aim of this paper is to provide an overview and summary of educators’ ideas and plans for educating the health workforce on the use of AI in health systems and services as a basis for strategic planning, investment, and further research in this area.

## Methods

### Overview

We used an expert survey method to gather information about AI education for the Australian health workforce. For the purpose of this work, we followed the Australian Institute of Health and Welfare (2019) definition to scope the health workforce [17], covering the 15 kinds of health care practitioners registered by the Australian Health Practitioner Regulation Agency (AHPRA) Boards, as well as other health care professionals (eg, audiologists and speech pathologists). Other professionals such as health administration and support workers (eg, health information managers, health technology suppliers, and health researchers) were also considered. We considered education that might be delivered in many contexts, such as a formal study program for entry into the workforce, continuing professional development endorsed as appropriate to maintain currency in the workforce, postbasic training leading to recognition as a specialist in the workforce, and an examination to certify competence as required to practice legally in the workforce.

### Participants

The intended participants were individuals who self-identified as having high-level expertise and experience in health workforce education and development; importantly, they were not required or expected to be experts in AI. They must indicate a relevant role that they held currently in a related organization for their responses to be included in this study; eligible roles included but were not limited to manager, coordinator, director, or committee chair (paid or unpaid) of an education or development portfolio. Respondents might hold such a role in more than one organization; they were not required to identify themselves or their organizational affiliations. The responses were expected to express the informed perspective of that individual and were not expected to be the official view of any organization. Although the exact number of potential respondents was unknown, the Alliance’s Workforce Working Group considered it feasible to reach at least 100 people.

### Survey Design

A 6-part survey was developed based on a scan of scholarly and gray literature about AI in health care. Our scan used search terms that occurred in combination in items published during the 2018-2020 period retrieved from Google Scholar and Google, representing the three intersecting fields of interest: AI (including expert systems, machine learning, and robotics), health (including medicine, nursing, allied health, and digital health), and education (including curriculum, teaching, and professional development). The sources of general relevance to our research are referenced in the *Introduction* section.

However, we found no existing question set suitable for our purpose, so we selected definitions and terms from recent authoritative sources. For example, we used a broad definition of AI that included machine learning, natural language processing, computer vision, and chatbots following the Academy of Medical Royal Colleges in the United Kingdom [18]. We derived a list of specific topics [19] and, for each topic, we provided a brief scope note for non-AI experts [19-29]. We derived a set of attitudes and beliefs from work by an internationally recognized advocate for professional development in the field of AI in medicine [30]. The survey was worded so that it could capture perspectives on AI education across professions and jurisdictions and allow for the expression of ideas about educating for organizational and technological change and social and global responsibility following the recommendations by Frenk et al [31]. The survey sections were (1) roles held in relevant types of organizations, (2) educational strategies and approaches in use or intended, (3) specific AI topics that are important in education and current content available, (4) learning activities and experiences that are important to support education, (5) attitudes and beliefs about AI that are important for education to address, and (6) additional comments.

Part 1 provided a list of relevant types of organizations and was the only section that was compulsory to complete; the others could be skipped over. Parts 1, 2, and 6 provided free-text response options. Parts 3, 4, and 5 provided 5-point Likert scale response options for 12-15 statements each plus free-text response options. The details of the survey items are presented in Multimedia Appendix 1.

The survey items were piloted with members of the Alliance’s Workforce Working Group. They found that the survey required approximately half an hour to complete; this was considered a possible deterrent but nevertheless an efficient way to elicit initial input on a complex topic from a range of educational experts.

### Data Collection and Analysis

A survey website and web-based form were created and tested using the Qualtrics (Qualtrics International, Inc) account of the University of Melbourne, and responses were monitored and summarized progressively during the period from October 2020 to December 2020 by GD. Recruitment occurred mainly through the Alliance’s electronic communication channels with members and partners, with periodic reminders and targeted follow-up messages to publicly listed contacts of major organizations such as the AHPRA Boards and professional colleges. Raw data were stored on a secure Qualtrics server in Australia.

Data were deidentified and aggregated, and distinctive written expressions were paraphrased so that no individual or organization would be readily identifiable. Descriptive statistical analysis of Likert scale data was performed using standard software in Qualtrics; the low number of responses did not warrant inferential analysis. Free-text data were thematically analyzed using grounded theory. Data were coded by an experienced qualitative analyst working independently (DC) and then reviewed jointly with another analyst (KG) until they reached an agreement on data interpretation and representation. A detailed initial report on quantitative and qualitative data was reviewed and critiqued by a meeting of the Alliance’s Workforce Working Group before the data were summarized further for publication.

### Ethics Approval

This study received Human Research Ethics approval (2056392.1) from the University of Melbourne.

## Results

A total of 103 people accessed the survey website as recorded by their responses to a verification question, of which 81 (78.6%) proceeded through the participant information home page and a consent form webpage and clicked on the *start survey* button. Of those 81 participants, 39 (48%) completed part 1, 29 (36%) completed part 2, 25 (31%) completed part 3, 23 (28%) completed part 4, 23 (28%) completed part 5, and 15 (19%) completed part 6.

### Part 1: Educational Experts’ Focus Areas

Most of the 39 survey respondents who completed part 1 held senior education-related roles in one or more education and training organizations: 46% (18/39) had roles in universities or other government-registered training organizations, and 17% (7/39) had roles in unregistered professional or industry training providers. The next largest group of respondents (5/39, 13%) held senior education-related roles in government-registered health care provider organizations. Of the remaining respondents who specified an organization type, 5% (2/39) each were from a national accrediting body, an independent medical research institute, a professional association, or an industry association and 3% (1/39) each were from an unregulated accrediting body or health care provider. The details are summarized in Table 1.

**Table 1:** The organizations of the educational experts.

Type of organizations where the experts were based	Respondents, n (%)
Education and training provider within the scope of the Australian National Training System	18 (46)
Other education and training provider	7 (17)
Organization registered as a health care provider with the Australian Department of Health	5 (13)
National board that registers practitioners and students and accredits education programs within the scope of the Australian Health Practitioner Regulation Agency	2 (5)
Independent medical research institute	2 (5)
Professional association	2 (5)
Industry association	2 (5)
Organization type not otherwise specified	2 (5)
Other organization that certifies or accredits individuals or programs	1 (3)
Other organization that provides health care services	1 (3)

The health workforce of immediate focus for most survey respondents (31/39, 80%) was working health care practitioners, mainly those registered with AHPRA (respondents were involved in education of enrolled nurses, medical professionals, midwives, nurse practitioners, paramedics, podiatrists, physicians, and dental practitioners) and speech pathologists, a nonregulated group. Four other workforce subgroups were the main concern among 5% (2/39) and 10% (4/39) of respondents: nonprofessional aged care, disability care, and community care workers; health information communication technology and health informatics workers; health data scientists and biomedical researchers; and university students across the range of the health workforce.

### Part 2: Organizational Approaches to AI Education

There were 36% (29/81) of survey respondents who offered insights into strategic thinking about how the current and future health workforce will acquire knowledge and skills to work with AI in health care. They identified distinct enablers and barriers to implementing strategic actions. They also provided examples of activities being planned or underway in their organizations to support learning and development.

Organizational strategizing ranged across four stages: stage 0 (meaning not yet under consideration), stage 1 (meaning there was consideration, exploration, and planning), stage 2 (meaning implementation was being designed), and stage 3 (meaning implementation was occurring). In some quarters, no strategic thought was being given to workforce learning and development for AI in health care; an example of stage 0 was “This has not been a focus area at this point in time for this organisation.” An example of stage 1 was “We use accreditation reports from universities on how they are adapting their courses for the future and meetings with health care and government stakeholders.” Some consideration, exploration, and formal planning approaches were underway, formulating initial ideas about what AI education was relevant for the current and future workforce. Respondents described overarching strategic considerations such as the clinical currency of the workforce and the upskilling of the workforce to display excellence in care. Among the approaches in which they were involved, they reported horizon scanning, identifying future roles for the profession, aggregating feedback from staff about professional knowledge gaps with impacts on service delivery and outcomes, consulting with senior leadership, and reviewing government policies and sector literature. More formal planning that they reported included conducting a review of professional performance frameworks and accreditation reports from universities, establishing advisory groups, instigating future needs planning within committees, and meeting with government stakeholders, as well as referencing AI education in strategy and planning documents. An example of stage 2 was “The inclusion of AI/ML [artificial intelligence and machine learning]...in all courses to prepare graduates for the workforce of the future is of great strategic importance and strategies are in place to commence this work.” This stage was marked by educational design strategies such as planning to integrate AI topics in graduate coursework, taking AI in health care into account when conducting a curriculum review, and planning specific content for continuing professional development. An example of stage 3 was the implementation of “specialized courses, micro-credits, for medical school students.” Respondents at this stage gave examples of specific activities that organizations were undertaking. These included developing specialized training courses, publishing articles in member newsletters to create awareness, promoting AI and ethics education in health care, using emerging and new technology in the delivery of education, and using simulated training in education.

Seven themes emerged to describe key opportunities and enablers of these organizational strategies: mobilization of expertise, influential leadership, leveraging collaborations, expanding continuing professional development, higher education planning and programming, government drivers, and health service improvements. Respondents saw possibilities to access academic expertise on AI and its applications, establish links with and participate in networks of experts, and become involved in consultative forums. They perceived that the championing of AI education for the health workforce was facilitated by dynamic thinkers who hold senior roles within organizations and by instrumental health care and academic stakeholders who are rallying to have greater influence. They also reported that there were strategic opportunities available within their organizations; for instance, to forge cross-sector collaborations, leverage support from medical colleges for AI education, and capitalize on investments in AI hubs. A further suggestion was that AI education for the health workforce could be facilitated by advocating for health service improvements; in other words, for infrastructure and quality improvements that would give rise to better patient experiences and outcomes and increased productivity and economic benefits. Already existing requirements for continuing professional development were recognized as enablers; continuing professional development initiatives around AI could upskill people in the workforce, including those in clinical and supervisory roles. Similarly, survey respondents looked to find ways through higher education planning and programming to use university resources, invest in academic teaching in the area, integrate AI applications in the design of instructional delivery, and comprehensively review courses so as to implement AI education within them. Respondents thought that government policy development, endorsement, and support were important drivers of AI education for the health workforce; for example, “a strong desire from the Health Department as a policy maker, funder and implementer.”

There were three major kinds of challenges and barriers to these organizational strategies: the lack of governance structures and processes, resource constraints, and cultural unreadiness. Respondents described ambiguity about the roles of workforce organizations and government departments in AI education for the health workforce and no clarity around processes for further investment in this area; a respondent said that “whilst we track and identify and amplify emerging issues...it still needs government regulation and funding to make the changes called for.” Resource constraints consisted of interrelated human, time, and funding issues. Human resource challenges were identified as “lack of a dedicated workforce” and limitations in expertise in the form of “not having a strong background in technology in healthcare” and “access to skills and consultants.” Time issues commented on were “competing priorities” and “curriculum already over-burdened.” Funding constraints were described as “limited university resources, poor external support from government,” “funding to do meaningful research and ongoing education,” and “funding to access and deploy technological solutions.” In terms of cultural unreadiness for AI education, respondents described “a challenge as we bridge the gap between the early and late adopters”; “a combination of resisting everything new, lack of knowledge in the area and complexity of implementing in an already over-stuffed curriculum”; and “compartmentalisation of the educational program.”

Activities that respondents reported preparing or implementing in their organizations to advance workforce knowledge and skill building regarding AI included auditing their current resources and skills, engaging stakeholders and developing research programs to improve engagement, conducting expert and stakeholder focus groups to identify growth opportunities, mobilizing capability and potential collaborations, providing relevant work placements, scoping current courses and qualifications available, and doing a gap analysis. Specific examples included seeking “high level advice on workforce future needs, and ways in which new course structures can enable that”; developing “surveys, prompt cards, reference videos, face-to-face training sessions, reference manuals, escalation plans”; proposing “short, non-compulsory, advanced courses for medical under- and post-graduates”; and using AI-enabled web-based learning platforms.

### Part 3: AI Educational Content and Provision

**Figure 1:**
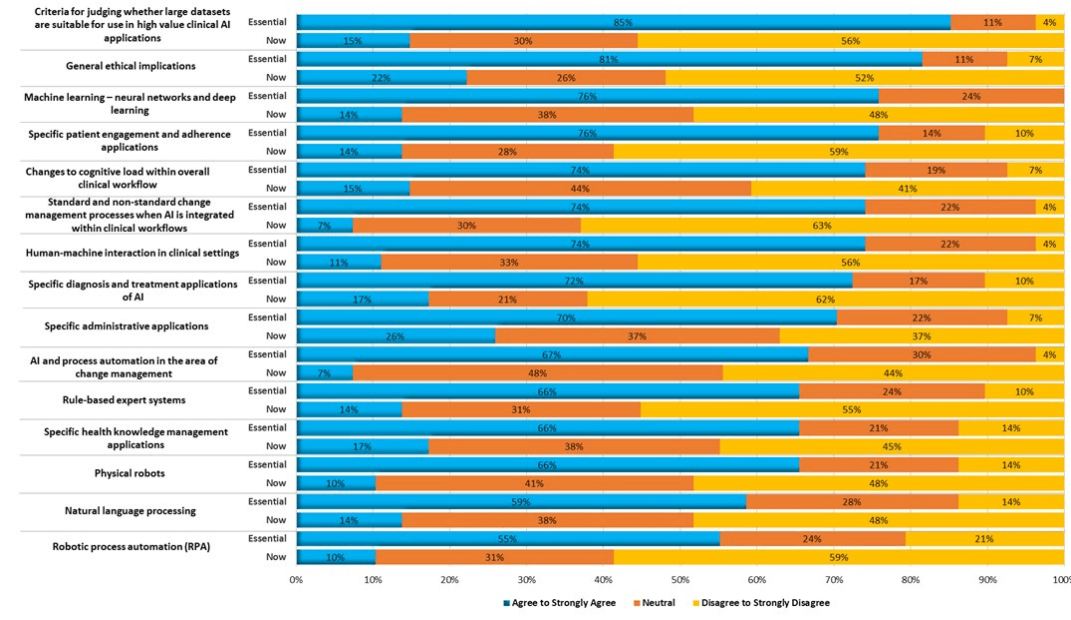
Artificial intelligence topics essential to teach and now taught. AI: artificial intelligence.

Approximately 31% (25/81) of survey respondents ranked a list of specific topics that could potentially be considered essential for health workforce competence in the next decade and judged whether the related education available to the health workforce at the time of the survey was sufficient to meet the need. Figure 1 shows the topics deemed essential, juxtaposed with the adjudged adequacy of the education being provided. Discrepancies between the importance of a topic and the adequacy of current education provision were consistently observed across all topics. Most respondents agreed or strongly agreed on the importance of three essential topics: criteria for judging whether large data sets are suitable for use in high-value clinical AI applications (20/25, 81%), general ethical implications (20/25, 81%), and machine learning (19/81, 76%). In contrast, only 15% (4/25), 22% (6/25), and 14% (4/25) of respondents agreed that these 3 topics were being taught adequately, respectively. This contrast was apparent even in the two topics that were ranked lowest although still considered essential by over half of respondents—natural language processing (15/25, 59%) and robotic process automation (14/25, 55%)—and only 14% (4/25) and 10% (3/25) of respondents thought that current education provision was adequate on each topic, respectively.

Table 2 synthesizes and paraphrases the respondents’ free-text comments elaborating on this part of the survey, with a few particularly pointed remarks quoted in full. The respondents also outlined additional AI education topics that they considered essential related to privacy, data security, product evaluation (including knowledge of adverse events), data accuracy, and representativeness of data sets for the Australian context. Additional importance was placed on “explainability, ... education in AI/ML programming using open source tools, AI governance skills,” and preparing the workforce for “what to do when AI stops working.”

**Table 2:** Comments on artificial intelligence (AI) educational content.

Topics	Key themes and remarks^a^
Criteria for judging whether large data sets are suitable for use in high-value clinical AI applications	Need for understanding the use of algorithms in machine learning Limited access to informational resources for current students enrolled in clinical degrees Useful and relevant topic for research training and participation
General ethical implications	Complex area Tailoring breadth and depth of training and informational tools would be warranted for different roles and contexts “This is a minefield area!”
Machine learning—neural networks and deep learning	Specific concepts to include the applications, implications, consequences, and limitations of using machine learning Focus on future needs Targeted and focused development of a group of individuals rather than the whole workforce
Specific patient engagement and adherence applications	Specialized training needs will be required depending on the care pathway, specific apps, and progress of these technologies Having established governance structures around value-based health care to complement education Changes to cognitive load within overall clinical workflow Tailoring levels of educational uptake for different disciplines “Models of care using these tools need to be built and clinically governed”
Changes to cognitive load within overall clinical workflow	Tailoring levels of educational uptake for different disciplines
Change management processes when AI is integrated within clinical workflows	Not currently part of education for health care practitioners “All of our training is still delivered face-to-face”
Human-machine interaction in clinical settings	Lack of informational access to this topic for current students in clinical degrees This topic could be reframed as part of ethical issue training
Specific diagnosis and treatment applications of AI	Might not be relevant to certain workforce roles End users of specific diagnosis and treatment applications of AI might not require in-depth specialist training and education This area will need to evolve to meet future needs (ie, development of standards and clinical governance regarding skill competencies)
Specific administrative applications	Area of interest given that a workforce competent in specific administrative applications would bring about productivity and clinical quality benefits Digital and ICT^b^ specialist workforce will require knowledge of specific administrative applications; the health care workforce could contribute by providing clinical input in this area AI and process automation in the area of change management Inclusion of risk management strategies in education and training “Knowing about the very many near misses is more important for the purposes of refining AIML^c^ than critical incidents alone”
Rule-based expert systems	Area of great potential and benefit (ie, reduction in cognitive load errors in emergency and intensive care settings) Further analysis required to understand the health workforce’s receptivity toward using rule-based expert systems and the implications for clinical practice in the next decade Specific health knowledge management applications
Specific health knowledge management applications	Limited access to resources (ie, databases) Participation in research projects was a way to promote learning
Physical robots	Highly relevant to medical professionals, nursing, aged care, and allied health A lack of clarity around the implications of using physical robots in clinical practice (ie, concrete examples would be required to understand how health workforce job roles might interact with physical robots) The need for training to be value-adding to ensure that physical robots improve and do not hinder health care workflow “Doctors probably learn more about robots from their kids’ toys than from their training.”
NLP^d^	Lack of access to real-world health data to teach learners about using algorithms; limited number of education opportunities and digital health literacy resources to support learning The clinical workforce might only require a general understanding of how NLP tools work, its applications, limitations, and consequences of use in health care Expertise of NLP specialists could be leveraged
RPA^e^	Important for the digital and ICT workforce to acquire skills in this area to support the health workforce in automating processes Health workforce could benefit from greater knowledge of ways to identify opportunities to apply RPA.

^a^Pointed remarks are in quotes.

^b^ICT: information and communication technology.

^c^AIML: artificial intelligence and machine learning.

^d^NLP: natural language processing.

^e^RPA: robotic process automation.

### Part 4: AI Educational Methods

**Figure 2:**
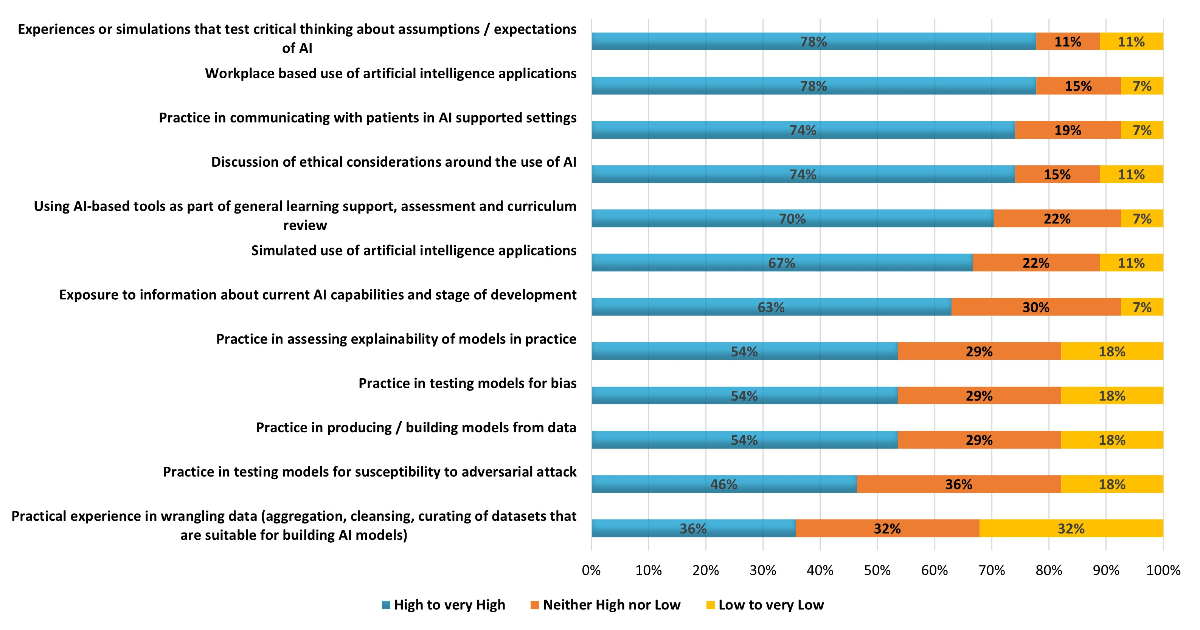
Value of different educational methods to teach artificial intelligence (AI).

Approximately 19% (23/81) of survey respondents addressed a set of 12 statements describing educational learning activities that could be used to build knowledge and skills for working with AI in health care and rated them according to the value they perceived each method to have (Figure 2). In total, 6 methods of learning were thought to be highly or very highly valuable by two-third (15/23, 67%) to three-quarter (17/23, 74%) of respondents, whereas only two methods were thought valuable by fewer than half of all respondents; namely, practice in testing models for vulnerability (11/23, 46%) and practice in wrangling data (8/23, 36%).

### Part 5: Education Regarding AI Attitudes and Beliefs

**Figure 3:**
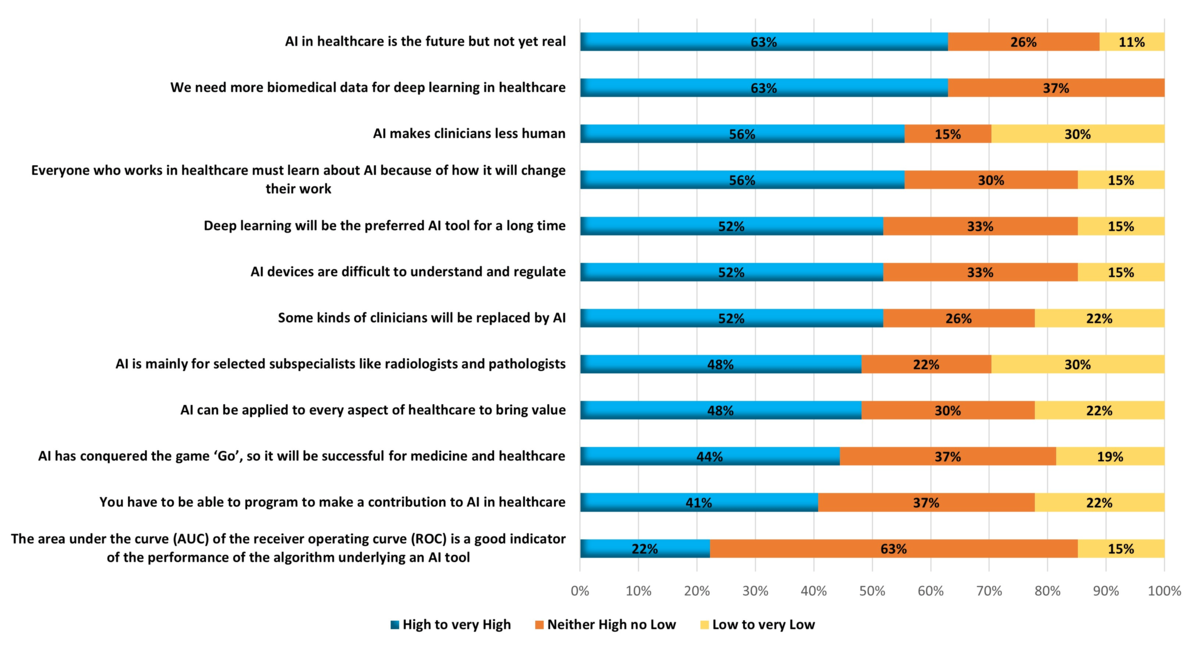
Importance of education to address attitudes and beliefs about artificial intelligence (AI).

Approximately 28% (23/81) of survey respondents considered a set of 12 statements describing poorly informed but widely held attitudes and beliefs about AI and rated them according to the importance of addressing them in AI education (Figure 3). A total of 7 attitudes were considered highly or very highly necessary to address in education by most respondents (between 12/23, 52% and 14/23, 63%). In total, 63% (14/23) of respondents were undecided about the following statement—*The area under the curve (AUC) of the receiver operating curve (ROC) is a good indicator of the performance of the algorithm underlying an AI tool*—with fewer than one-quarter (5/23, 22%) rating it important for education to address this belief.

## Discussion

### Principal Findings

This survey gathered detailed observations about AI education from a range of experts in senior roles associated with health workforce training and professional development. Their thought leadership reflects an array of mainstream health care professions as well as important groups who are ancillary to frontline health care workers. Responses to this survey showed that many different health workforce subgroups and interest groups have a stake in education and professional development on AI in health care; this is not only of concern in selected medical specializations but also in fields ranging from aged care to speech pathology. As this study reached throughout a national health system and elicited responses from a wide range of senior stakeholders and influencers in health workforce education and professional development, it makes a distinctive contribution compared with much of the previously published work on this topic, which reflects expert perspectives of individuals or narrowly defined workforce subgroups.

The development of the survey instrument was informed by a multifaceted view of the curriculum, so it elicited ideas not only about what should be learned (topics) but also how (learning activities), why (attitudes and beliefs), and the mechanisms helping and hindering educational change (strategic actions). Basing the survey around accepted concepts and definitions of AI in health care from published literature aided in clarifying priorities and avoided overlooking major aspects of AI education for the health workforce. Furthermore, the content of the survey itself may have served an informational, informal learning purpose for some of the people who started but did not complete it. By increasing their awareness of AI in health care, the survey project may have prompted some respondents to give further attention to this immature area of health workforce education and professional development. The design of the survey is such that it may be adapted and reused in other settings and could support longitudinal research over time as this aspect of workforce education and development matures.

Responses showed that the health sector is broadly in agreement but, on the whole, has not progressed far in plans to address this workforce need. In some quarters, it is possibly misdirected. For example, some responses conflated AI with health data analytics and digital health generally, which would dilute the deeper understanding of the implications of AI; some of the strategies proposed using AI-supported teaching techniques and learning analytics tools, which per se would not lead to a deeper understanding of AI-supported health care. Many varied opportunities and enablers of action were identified, suggesting optimism about the ability to make progress on this area of workforce education; the systemic challenges and barriers mentioned were fewer, although they presented substantial roadblocks.

The results also highlighted the priorities for education to address social and technical facets of AI, including ethical implications, suitability of large data sets for use in AI clinical applications, principles of machine learning, and specific diagnosis and treatment applications of AI as well as alterations to cognitive load during clinical work and the interaction between humans and machines in clinical settings. Although identification of priorities represents a first step, there are many activities such as capability building among educators and competency-based content development that are needed before implementation can occur at scale in health workforce education. The respondents also outlined barriers to implementation, which included a lack of governance structures and processes, resource constraints, and cultural adjustment. These are ubiquitous and represent major challenges; they are not specific to education in AI but are known to affect other areas, too, such as education to prepare the workforce for digital health generally [32].

Among the responses were almost no examples of educational resources or approaches that would build or benchmark competencies to work in interprofessional health care settings or in international contexts. Having noted this, resource constraints and development costs may be ameliorated if educators are aware of a number of web-based resources already in existence. The Australian eHealth Research Centre has compiled a series of real-world use cases of AI in health care for public information and education [33]. Around the world, a selective list of useful foundations for workforce education and development includes the Coursera AI course catalogue [34], the collection of readings on machine learning and AI on Medium [35], the AI Adventures playlist of Google Cloud Tech [36], a UK National Health Service and University of Manchester interprofessional course on AI for health care [37], and a certification examination and supporting learning and professional development overseen by a multidisciplinary advisory group of domain experts in the American Board of Artificial Intelligence in Medicine [38].

### Limitations

Although the results reflected a cross-section of professions, organizations, and jurisdictions, they do not have statistical power. It was not possible to calculate a participation rate; overall, web-based access numbers met the minimum expectations of the potential reach of the survey, but completion numbers fell short. The survey required complex responses from a group of respondents known to be time-poor; therefore, survey fatigue likely accounted for some of the difference between the number who started and the number who completed all sections. Nevertheless, this was the most efficient method available to the researchers to begin a national interprofessional investigation, and it provides material that can be used in follow-up workshops for subgroups of those targeted in the survey.

Furthermore, the survey yielded valuable qualitative data; the free-text responses were thoughtful and extensive, and they provide a consolidated view of Australian educational experts’ observations and aspirations regarding AI education for the health workforce. This study was the first of its kind and not only in Australia; however, it is only a first step in work toward education and professional development on AI that is delivered efficiently to the whole health workforce as well as tailored carefully for different roles and responsibilities within it.

### Conclusions

This survey provides a baseline for further work by those responsible for enabling the health workforce as the optimization and ramifications of AI in health care unfold. These are early days in supporting the current and future health workforce to be able to work safely and effectively with AI, but the situation is evolving rapidly. There are calls for this work to proceed in partnership between education providers and AI technology providers to ensure that uniform training is available across health care subgroups and jurisdictions [39]. The methodology used to design and conduct this survey can be adapted for use in other health systems beyond Australia. From other areas of education on health informatics and digital health, we know that some topics and questions will be of global interest and concern, whereas other topics and questions will need to be customized to the distinctive social, political, and technical contexts of particular regional and national health care systems. Wider administration of surveys such as this one and detailed work to address the priority learning needs will assist educators and education authorities around the world who are responsible for preparing the health workforce to minimize the risks and realize the benefits of implementing AI in health care.

## Appendix

Multimedia Appendix 1 Survey items.

## Abbreviations

AHPRA: Australian Health Practitioner Regulation Agency
AI: artificial intelligence
