# Supplementary material for: Artificial Intelligence Education for the Health Workforce: Expert Survey of Approaches and Needs
Source: JMIR Med Educ. 2022 Apr 4;8(2):e35223. doi: 10.2196/35223 (PMC9016514; doi:10.2196/35223)
Supplement: Multimedia Appendix 1 [file mededu_v8i2e35223_app1.docx]

## Appendix A

**Survey items.** Artificial intelligence education for the Australian health workforce

**1. ROLE AND ORGANISATION**

1.1 What is the **main aim** of the health-related organisation where you hold a senior education-related position? (drop down list)

1. Education and training provider within the scope of the Australian National Training System (e.g. health and IT schools within universities and other registered training organisations)
2. Education and training provider not within the scope of the Australian National Training System (e.g. profession and industry training providers not included in A)
3. National Board that registers practitioners and students and accredits education programs within the scope of the Australian Health Practitioner Regulation Agency (e.g. Medical Board of Australian and all other health profession boards listed at https://www.ahpra.gov.au/National-Boards.aspx; and their delegated authorities, e.g. Australian Medical Council, Optometry Council of ANZ, etc.)
4. Organisation that certifies individuals’ competencies or accredits education providers not within the scope of the Australian Health Practitioner Regulation Agency (e.g. Australasian College of Health Service Management, Health Information Management Association of Australia, etc.)
5. Organisation registered as a Healthcare Provider Identifier-Organisation (HPI-O) with the Australian Department of Health (e.g. hospitals, private clinics, primary care providers and others, as explained at https://meteor.aihw.gov.au/content/index.phtml/itemId/426830)
6. Organisation that delivers healthcare services not identified as a Healthcare Provider Identifier-Organisation (HPI-O) by the Australian Department of Health (e.g. residential aged care providers, primary health networks)
7. Independent medical research institute given Administering Institution status by the National Health and Medical Research Council (list at https://www.nhmrc.gov.au/funding/manage-your-funding/nhmrcs-administering-institutions)
8. Professional association within the Australian Bureau of Statistics definition of Professionals (e.g. Australasian Health and Research Data Manager's Association)
9. Industry association (e.g. Medical Software Industry Association)
10. Government agency not covered above
11. Registered business not covered above
12. Not-for-profit organisation not covered above
13. Other organisation type

1.2 Please tell us about the dimensions of **the workforce** that is of direct concern to your organisation.

1.2.1 Brief description of this workforce: [Open comment]

(e.g. paramedics in Australia; pharmacy students in Queensland; data scientists in Sydney)

1.2.2 Estimated number of people in this workforce at present: [Scale 1 to 1M]

1.2.3 Estimated number currently in training to enter this workforce: [Scale 1 to 1M]

1.2.4 Estimated number expected to be in this workforce in 2025: [Scale 1 to 1M]

1.2.5 Total years of post-secondary education and training required to enter this workforce: [Scale 1 year to 20 years]

1.3 What is your role in this organisation? [Open comment]

**2 CURRENT STRATEGIES RE AI EDUCATION**

2.1  At a strategic level, what consideration are you giving to how the current and /or future health workforce will acquire knowledge and skills for AI in healthcare?

(For example, indicate directions that your organisation is likely to take, if possible provide a high-level summary of planning meetings, identify references in planning documents, etc.)

[Space for open comments]

2.2  What are the main opportunities or enablers that you have available, to implement strategy in this area?

[Space for open comments]

2.3 What are the main challenges or barriers that you face, in implementing strategy in this area?

(For example, access to skills, 457 visa limitations, restrictions on engaging external consultants, hiring freezes for staff, etc.)

[Space for open comments]

2.4  If you are implementing an approach to workforce knowledge and skill building , describe what actions you are taking.

(For example, briefly describe activities, resources, methods, tools that you are using or preparing to use to support learning and development)

[Space for open comments]

**3 YOUR PERSPECTIVE ABOUT SPECIFIC TOPICS IN AI EDUCATION**

What emphasis would you place on the topics listed here, derived in part from (4), in educating the health workforce? Please respond to both statements about each topic. Use spaces to give specific examples to support your responses.

Key: A=Agree D=Disagree N=Neither agree nor disagree S=Strongly

3.1 Machine learning – neural networks and deep learning

A short definition from https://hackernoon.com/deep-learning-vs-machine-learning-a-simple-explanation-47405b3eef08 Machine learning is a subset of artificial intelligence involved with the creation of algorithms which can modify themselves without human intervention to produce desired output by feeding itself through structured data. Deep Learning is a subset of machine learning where algorithms are created and function similar to those in machine learning, but there are numerous layers of these algorithms- each providing a different interpretation to the data it feeds on. Such a network of algorithms are called artificial neural networks, so named as their functioning is inspired by the function of the human neural networks present in the brain.

3.1.1 Education and training is essential for health workforce competence in the next decade.

SA A N D SD

3.1.2 Education and training available now is adequate to meet health workforce needs.

SA A N D SD

3.1.3 [Space for open comment]

3.2 Natural language processing

A short definition from https://en.wikipedia.org/wiki/Natural_language_processing NLP is a subfield of linguistics, computer science, information engineering, and artificial intelligence concerned with the interactions between computers and human (natural) languages, in particular how to program computers to process and analyze large amounts of natural language data.

3.2.1 Education and training is essential for health workforce competence in the next decade.

SA A N D SD

3.2.2 Education and training available now is adequate to meet health workforce needs.

SA A N D SD

3.2.3 [Space for open comment]

3.3 Rule-based expert systems

A short definition from https://papers.ssrn.com/sol3/papers.cfm?abstract_id=3082734 An expert system is computer program that emulates the behavior of a human expert. The expert system represents knowledge solicited from human experts as data or production rules within a computer program. These rules and data can be used to solve complex problems.

3.3.1 Education and training is essential for health workforce competence in the next decade.

SA A N D SD

3.3.2 Education and training available now is adequate to meet health workforce needs.

SA A N D SD

3.3.3 [Space for open comment]

3.4 Physical robots

A short definition from https://en.wikipedia.org/wiki/Robot The word robot can refer to both physical robots and virtual software agents, but the latter are usually referred to as bots. Robots tend to possess some or all of the following abilities and functions: accept electronic programming, process data or physical perceptions electronically, operate autonomously to some degree, move around, operate physical parts of itself or physical processes, sense and manipulate their environment, and exhibit intelligent behavior, especially behavior which mimics humans or other animals

3.4.1 Education and training is essential for health workforce competence in the next decade.

SA A N D SD

3.4.2 Education and training is available now to meet health workforce needs to become competent.

SA A N D SD

3.4.3 [Space for open comment]

3.5 Robotic process automation (RPA)

A short definition from https://internetofthingsagenda.techtarget.com/definition/robotic-process-automation. RPA is the use of software with artificial intelligence (AI) and machine learning capabilities to handle high-volume, repeatable tasks that previously required humans to perform.

3.5.1 Education and training is essential for health workforce competence in the next decade.

SA A N D SD

3.5.2 Education and training available now is adequate to meet health workforce needs.

SA A N D SD

3.5.3 [Space for open comment]

3.6 Specific diagnosis and treatment applications of AI

For example, through radiological image analysis, or analysis of other types of images such as retinal scanning or genomic-based precision medicine (from https://www.ncbi.nlm.nih.gov/pmc/articles/PMC6616181/)

3.6.1 Education and training is essential for health workforce competence in the next decade.

SA A N D SD

3.6.2 Education and training available now is adequate to meet health workforce needs.

SA A N D SD

3.6.3 [Space for open comment]

3.7 Specific patient engagement and adherence applications

For example, using app-based smartphone technology, coupled with case management, to ensure that orally delivered medications are taken according to schedule (from https://www.healthcareitnews.com/ai-powered-healthcare/provider-taps-ai-powered-app-improve-medication-adherence)

3.7.1 Education and training is essential for health workforce competence in the next decade.

SA A N D SD

3.7.2 Education and training available now is adequate to meet health workforce needs.

SA A N D SD

3.7.3 [Space for open comment]

3.8 Specific health knowledge management applications

For example, https://quertle.com/products/qinsight/ which speeds up and improves results of searching and summarising biomedical literature.

3.8.1 Education and training is essential for health workforce competence in the next decade.

SA A N D SD

3.8.2 Education and training available now is adequate to meet health workforce needs.

SA A N D SD

3.8.3 [Space for open comment]

3.9 Specific administrative applications

For example, robotic process automation of claims processing, clinical documentation, revenue cycle management and medical records management (from https://www.ncbi.nlm.nih.gov/pmc/articles/PMC6616181/)

3.9.1 Education and training is essential for health workforce competence in the next decade.

SA A N D SD [

3.9.2 Education and training available now is adequate to meet health workforce needs.

SA A N D SD

3.9.3 [Space for open comment]

3.10 General ethical implications

For example, from https://www.ncbi.nlm.nih.gov/pmc/articles/PMC6616181/ there are likely to be incidents in which patients receive medical information from AI systems that they would prefer to receive from an empathetic clinician.

3.10.1 Education and training is essential for health workforce competence in the next decade.

SA A N D SD

3.10.2 Education and training available now is adequate to meet health workforce needs.

SA A N D SD

3.10.3 [Space for open comment]

3.11 Criteria for judging whether large datasets are suitable for use in high value clinical AI applications

For example, as explained in https://www.ncbi.nlm.nih.gov/pmc/articles/PMC6616181/ Machine learning systems in healthcare may also be subject to algorithmic bias, perhaps predicting greater likelihood of disease on the basis of gender or race when those are not actually causal factors.

3.11.1 Education and training is essential for health workforce competence in the next decade.

SA A N D SD [

3.11.2 Education and training available now is adequate to meet health workforce needs.

SA A N D SD

3.11.3 [Space for open comment]

3.12 Human-machine interaction in clinical settings

For example, as explained in https://www.ncbi.nlm.nih.gov/pmc/articles/PMC6616181/ when mistakes are made by AI systems in patient diagnosis and treatment it may be difficult to establish accountability for them.

3.12.1 Education and training is essential for health workforce competence in the next decade.

SA A N D SD

3.12.3 Education and training available now is adequate to meet health workforce needs.

SA A N D SD

3.12.3[Space for open comment]

3.13 Standard and non-standard change management processes when AI is integrated within clinical workflows

For example, from https://healthitanalytics.com/features/5-steps-for-planning-a-healthcare-artificial-intelligence-project, training your people, engaging them to use the tools correctly, optimizing the workflows.

3.13.1 Education and training is essential for health workforce competence in the next decade.

SA A N D SD

3.13.2 Education and training available now is adequate to meet health workforce needs.

SA A N D SD

3.13.3 [Space for open comment]

3.14 AI and process automation in the area of change management

For example, automating enterprise application monitoring activities, to head off critical incidents before they occur and to reduce the need for manual intervention (from https://www.ibm.com/downloads/cas/QAQMRGVN)

3.14.1 Education and training is essential for health workforce competence in the next decade.

SA A N D SD

3.14.2 Education and training available now is adequate to meet health workforce needs.

SA A N D SD

3.14.3 [Space for open comment]

3.15 Changes to cognitive load within overall clinical workflow

For example, from https://www.nature.com/articles/s41746-019-0190-1 automating clinical documentation with digital scribes, using speech recognition to eliminate manual documentation by clinicians

3.15.1 Education and training is essential for health workforce competence in the next decade.

SA A N D SD

3.15.2 Education and training available now is adequate to meet health workforce needs.

SA A N D SD

3.15.3 [Space for open comment]

3.17 Other AI related topics where you consider that education and training is essential

[space to provide details]

3.18 Other AI related topics where you know that education and training is available

[space to provide details]

**4 YOUR PERSPECTIVE ON AI EDUCATIONAL EXPERIENCES**

There are many possible methods for supporting learning about AI. Please rate the value that you see in providing education by the following methods or means, for the purpose of building knowledge and skill to work with AI in healthcare:

4.1 workplace based use of artificial intelligence applications

Very low Low Neither high nor low High Very high

4.2 simulated use of artificial intelligence applications

Very low Low Neither high nor low High Very high

4.3 experiences or simulations that test critical thinking about assumptions / expectations of AI

Very low Low Neither high nor low High Very high

4.4 discussion of ethical considerations around the use of AI

Very low Low Neither high nor low High Very high

4.5 exposure to information about current AI capabilities and stage of development

Very low Low Neither high nor low High Very high

4.6 practice in communicating with patients in AI supported settings

Very low Low Neither high nor low High Very high

4.7 using AI-based tools as part of general learning support, assessment and curriculum review

Very low Low Neither high nor low High Very high

4.8 practical experience in wrangling data (aggregation, cleansing, curating of datasets that are suitable for building AI models)

Very low Low Neither high nor low High Very high

4.9 practice in producing / building models from data

Very low Low Neither high nor low High Very high

4.10 practice in testing models for bias

Very low Low Neither high nor low High Very high

4.11 practice in testing models for susceptibility to adversarial attack

Very low Low Neither high nor low High Very high

4.12 practice in assessing explainability of models in practice

Very low Low Neither high nor low High Very high

4.13 Other education methods that you consider valuable for this purpose

[space to provide details]

**5 YOUR PERSPECTIVE ON EDUCATION ABOUT AI ATTITUDES AND BELIEFS**

Should workforce education explicitly address attitudes and beliefs that have been expressed previously about AI in healthcare, such as those listed here (3)? Please rate the importance you place on educating the workforce to think critically about each statement:

51 AI has conquered the game ‘Go’, so it will be successful for medicine and healthcare.

Very low Low Neither high nor low High Very high

5.2 AI can be applied to every aspect of healthcare to bring value.

Very low Low Neither high nor low High Very high

5.3 Everyone who works in healthcare must learn about AI because of how it will change their work.

Very low Low Neither high nor low High Very high

5.4 AI is mainly for selected subspecialists like radiologists and pathologists.

Very low Low Neither high nor low High Very high

5.5 Some kinds of clinicians will be replaced by AI.

Very low Low Neither high nor low High Very high

5.6 AI makes clinicians less human.

Very low Low Neither high nor low High Very high

5. 7 AI devices are difficult to understand and regulate.

Very low Low Neither high nor low High Very high

5.8 Deep learning will be the preferred AI tool for a long time.

Very low Low Neither high nor low High Very high

5.9 We need more biomedical data for deep learning in healthcare.

Very low Low Neither high nor low High Very high

5.10 The area under the curve (AUC) of the receiver operating curve (ROC) is a good indicator of the performance of the algorithm underlying an AI tool.

Very low Low Neither high nor low High Very high

5.11 You have to be able to program to make a contribution to AI in healthcare.

Very low Low Neither high nor low High Very high

5.12 AI in healthcare is the future but not yet real.

Very low Low Neither high nor low High Very high

5.13 Other attitudes and beliefs about AI which are important for education to address

[space to provide details]

**6 ADDITIONAL DETAILS**

6.1 If you would you like to make any other comments about AI education for the health workforce, please use the space here.

[open comment]

6.2 If you would be willing to be contacted by a researcher to discuss your responses to this survey, by telephone or teleconference when convenient to you, please insert your email address here.

[open comment]

6.3 If you would you like to be notified when the findings of this survey are made public, please insert your email address here.

[open comment]
